# First-Principles Modeling of Charge Transfer Excitons in Organic Solar Cells

**DOI:** 10.1021/acs.jctc.6c01456

**Published:** 2026-10-05

**Authors:** Vishal Jindal, Michael J. Janik, Scott T. Milner

**Affiliations:** Department of Chemical Engineering, 311285 The Pennsylvania State University, University Park, Pennsylvania 16802, United States

## Abstract

Charge-transfer (CT) excitons formed at donor–acceptor interfaces play a central role in charge separation in organic solar cells. Their binding energy and spatial structure are highly sensitive to local molecular geometry, particularly in disordered morphologies. In this work, we present a multiscale computational framework to model CT excitons at the interface between poly­(3-hexylthiophene) (P3HT) and the nonfullerene acceptor O-IDTBR, a representative polymer-nonfullerene blend. We extend a previously developed tight-binding model to include kinetic energy, Coulomb interaction and dielectric polarization, with all parameters derived from density functional theory (DFT). Using molecular dynamics simulations of an amorphous P3HT:IDTBR blend, we identify hundreds of donor–acceptor contact geometries and compute the CT exciton for each by minimizing the total energy with respect to electron and hole wavefunctions. We analyze the distribution of CT exciton energies, compare them with localized excitons on IDTBR, and estimate binding energies relative to free polarons in the bulk phases. By decomposing the CT energy into one-body kinetic and two-body (Coulomb + polarization) contributions, we show that the primary variation of CT energies is controlled by the underlying polaron energy landscape, while configuration-dependent Coulomb and polarization stabilization explains the residual scatter and distinguishes true CT states from quasi-separated polarons. Additionally, we place these results in the context of a full energy landscape from HOMO–LUMO gaps to excitons, CT states, and polarons, highlighting how interfacial structure tunes the balance between exciton formation and dissociation. This approach enables predictive evaluation with no adjustable parameters of CT excitons across realistic morphologies, and offers design principles for engineering interfaces that promote efficient charge separation in organic photovoltaics.

## Graphical abstract

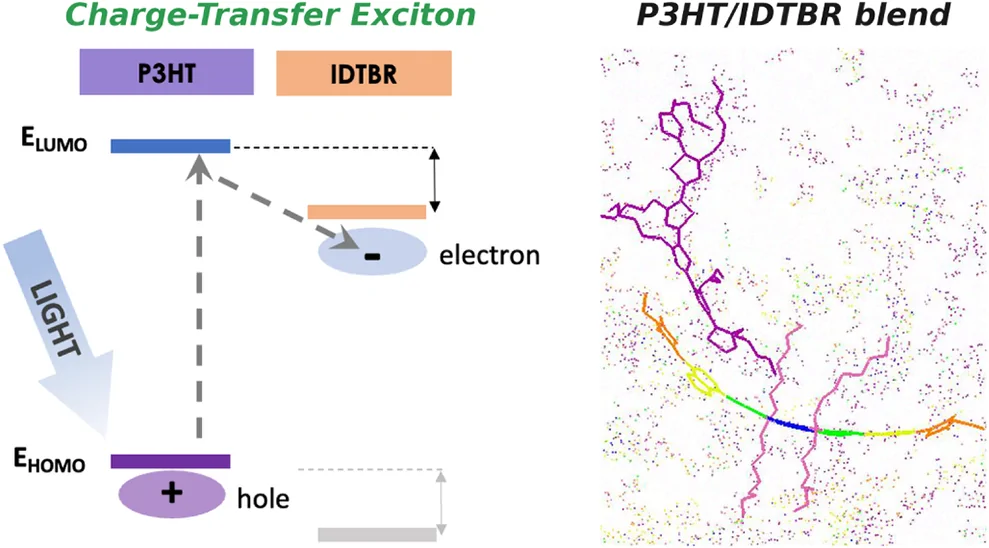

## 1 Introduction

Charge separation at donor–acceptor interfaces is fundamental to the function and efficiency of organic solar cells and optoelectronic devices.^1^ Upon photoexcitation, strongly bound singlet excitons are generated within the donor polymer or acceptor domains. These excitons migrate to donor–acceptor interfaces, where energy level offsets facilitate charge transfer. This leads to the formation of interfacial charge-transfer (CT) excitons, which are Coulombically bound pairs of a hole residing on a donor molecule and an electron residing on an acceptor molecule in close contact, at the donor–acceptor interface.^2^ From the CT state, electrons and holes may either dissociate to free carriers, or recombine and potentially decay radiatively or nonradiatively.

Charge-transfer excitons play a important role in determining the internal quantum efficiency, open-circuit voltage, and subgap absorption features of organic photovoltaic devices.^3–7^ Whether a CT exciton successfully dissociates into free carriers or recombines impacts the overall device performance.^8,9^ Consequently, the spatial structure and binding energy of CT states are critical determinants of charge separation efficiency, especially in nonfullerene acceptor systems that exhibit small donor–acceptor frontier orbital energy offsets.

Experimental signatures of CT excitons appear as weak subgap features in external quantum efficiency (EQE) and electroluminescence (EL) spectra.^8,10^ Sensitive techniques such as Fourier-transform photocurrent spectroscopy (FTPS), photothermal deflection spectroscopy, and highly sensitive EQE measurements have enabled detection of CT absorption and emission onsets.^11,12^ The optical energy of the CT state is typically estimated from the EQE spectrum by fitting it to a Marcus theory-based line shape, which accounts for the reorganization energy and energetic disorder.^5,13^ Binding energies are then inferred by comparing this CT energy with the interfacial donor–acceptor gap, as determined from ultraviolet photoelectron spectroscopy or cyclic voltammetry.^14–21^

Recent studies on polymer and nonfullerene acceptor (NFA) blends, such as P3HT-IDTBR, have shown that CT excitons can have binding energies with respect to free polarons as low as 0.1–0.3 eV, significantly lower than those found in fullerene-based systems.^11,22–25^ This reduced binding facilitates spontaneous charge separation at room temperature and is a key reason for the better performance of recent NFA-based organic solar cells.^26–29^ However, despite clear spectroscopic evidence of CT states, the spatial extent and detailed wavefunction structure of these excitons remain experimentally inaccessible. In particular, it is not yet clear which specific aspects of local chain conformation and donor–acceptor contact geometry control the distribution of CT energies and their ability to dissociate into free carriers.

Computational efforts to characterize CT excitons include time-dependent density functional theory (TD-DFT) and many-body perturbation theory (GW-BSE).^30–32^ While these methods can predict CT energies in donor–acceptor dimers, they are computationally expensive and sensitive to the choice of exchange-correlation functional or dielectric screening model.^33–35^ Moreover, they are typically limited to small systems in idealized geometries, and do not provide a transparent connection between molecular configuration and exciton delocalization.^2,36,37^

We adopt a tight-binding (TB) approach that extends earlier models developed by our group for polarons and excitons in conjugated polymers.^38–44^ This framework offers a physically transparent and computationally efficient means of modeling charge carriers and excitons in large, disordered systems. By parameterizing the TB Hamiltonian with onsite and hopping terms extracted from density functional theory (DFT) calculations, we preserve the accuracy of quantum chemistry while gaining the scalability of coarse-grained modeling.

We use the tight-binding model to describe CT excitons formed at interfaces between the donor polymer poly­(3-hexylthiophene-2,5-diyl) (P3HT), and the acceptor molecule (5Z,5′Z)-5,5′-((7,7′-(4,4,9,9-tetraoctyl-4,9-dihydro-s-indaceno­[1,2-b:5,6-b′]­dithiophene-2,7-diyl)­bis­(benzo­[c]­[1,2,5]­thiadiazole-7,4-diyl))­bis­(methanylylidene))­bis­(3-ethyl-2-thioxothiazolidin-4-one) (O-IDTBR). Although P3HT-IDTBR is no longer the most efficient donor–acceptor pair for organic solar cells, it is well studied experimentally and computationally, so we have chosen this system for the present study. However, the methods developed here can be applied to other molecular architectures.

The total CT exciton energy is decomposed into kinetic energy, Coulombic interaction between the electron and hole, and stabilization from the polarization of the surrounding dielectric medium. Our model explicitly describes the electron–hole wavefunction, which enables direct computation of spatial delocalization and binding energy. This decomposition also allows us to separate one-body kinetic energy contributions from two-body Coulomb and polarization effects, and to analyze how each term contributes to CT-energy heterogeneity at disordered interfaces.

Here, we focus on how structural heterogeneity at the donor–acceptor interface influences the energetics and dissociation behavior of CT excitons. By applying our tight-binding framework to hundreds of P3HT-IDTBR configurations extracted from molecular dynamics simulations, we compute the optimal CT exciton for each donor–acceptor contact without adjusting any electronic parameters. This allows us to quantify how variations in molecular geometry affect exciton localization, energy, and binding strength, and to identify when CT energetics are dominated by the one-body contribution versus two-body Coulomb and polarization interactions. We further compare the computed CT exciton energies with bulk exciton and polaron energies to construct a complete energy landscape, providing mechanistic insight into the charge separation pathway.

This paper is organized as follows. We first present the tight-binding Hamiltonian, parameterization scheme, and expressions for kinetic, Coulomb, and polarization contributions to the exciton energy. We then apply this framework to compute CT exciton properties across a diverse set of P3HT-IDTBR interfacial structures. We analyze the distribution of CT exciton energies, compare them with localized excitons on IDTBR, and quantify binding energies relative to free polarons in the bulk phases. Finally, we discuss these results in the context of a unified energy level diagram, highlighting how interfacial morphology modulates the depth and dissociation potential of CT states.

## 2 Methods

### 2.1 Tight-Binding Model

We describe the behavior of an electron or hole on a conjugated system using a tight-binding model, as developed in our previous work.^39,43–45^ The tight-binding model is a coarse-grained electronic structure approach in which the electronic degrees of freedom are reduced to a single frontier orbital per monomer, referred to as a site. A charge carrier can occupy these sites, each characterized by an onsite energy (ϵ), and can hop between neighboring sites via hopping matrix elements (*t*), enabling delocalization across the conjugated backbone.

The tight-binding Hamiltonian for a charge carrier along a conjugated system is expressed as

1 $$H=\overset{n}{\underset{k=1}{\sum }}\epsilon_{k}c_{k}^{†}c_{k}-\overset{n-1}{\underset{k=1}{\sum }}t_{k}\left(c_{k}^{†}c_{k+1}+c_{k+1}^{†}c_{k}\right)$$

Here, $c_{k}^{†}$ and *c*_*k*_ are the creation and annihilation operators for a charge carrier on site *k*; ϵ_*k*_ is the onsite energy at site *k*; and *t*_*k*_ is the hopping matrix element between sites *k* and *k* + 1. These parameters are obtained from density functional theory (DFT) calculations performed on constituent monomers and dimers, i.e., single aromatic moieties that constitute tight-binding sites, and bonded pairs of adjacent such moieties.^44^ In recent work, we applied this methodology to derive tight-binding parameters for disordered polymer morphologies and investigated charge transport in amorphous poly­(3-hexylthiophene-2,5-diyl) (P3HT) melts.^45^

In this study, we focus on modeling charge transfer excitons at the donor–acceptor interface between P3HT and O-IDTBR. P3HT is a widely used donor material, while O-IDTBR is a nonfullerene acceptor employed in organic photovoltaic devices. The molecular structure of O-IDTBR consists of an electron-rich indacenodithiophene (IDT) core flanked by electron-withdrawing benzothiadiazole (BT) and rhodanine groups. At the interface between amorphous P3HT and O-IDTBR, the most probable close contact occurs between the thiophene ring in P3HT and the BT ring in IDTBR.^46^

In our model, each aromatic moiety is treated as a site. These units are geometrically rigid and electronically well-coupled, such that their internal electronic structure is only weakly perturbed by conformational variations in the polymer chain and acceptor molecule. For example, a P3HT-IDTBR interface is modeled as an array of sites corresponding to monomers along the conjugated backbone: Ph, Th, BT and Rh (see Figure 1).

**Figure 1:**
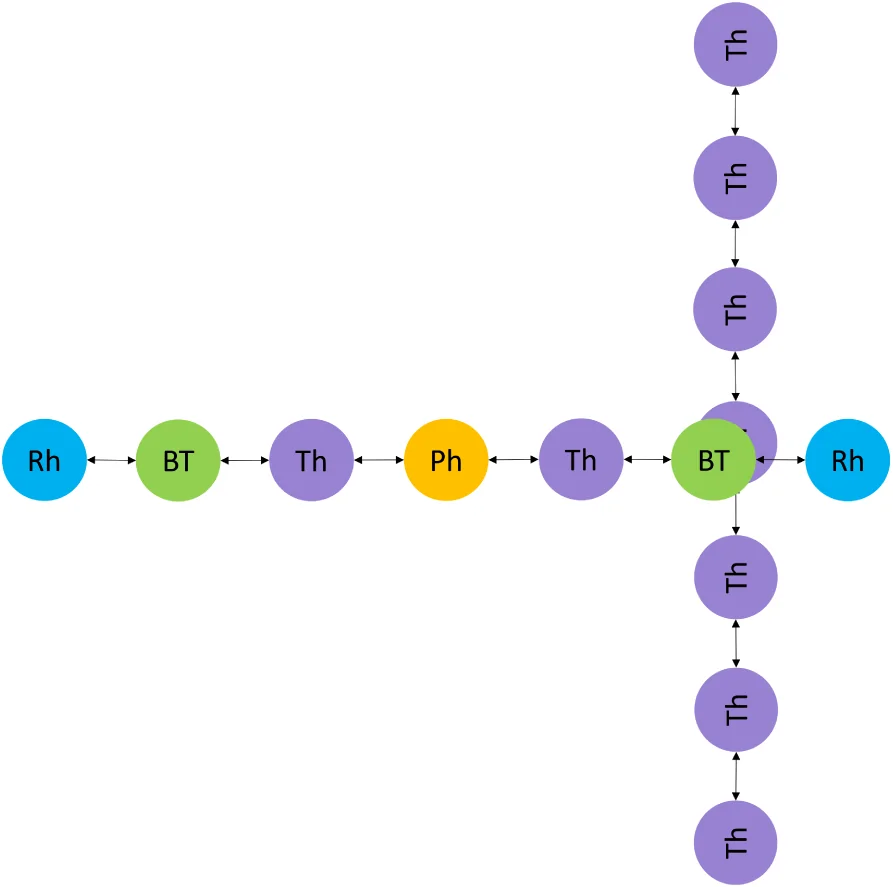
Schematic showing a close contact between P3HT and an IDTBR molecule. Constituent monomers are coarse-grained as sites (Ph—Phenylene, Th————Thiophene, BT—Benzothiadiazole, Rh—Rhodanine), interconnected either within the same molecule or across a close-contact interface.

Using the tight-binding model, we calculate the kinetic energies of spatially separated electrons and holes. In the charge-transfer (CT) state, the electron and hole remain bound by Coulomb attraction and interact with the surrounding dielectric medium.

### 2.2 Charge Transfer Exciton Energy

The total energy of a charge transfer (CT) exciton, *E*_*CT*_, is expressed as the sum of three contributions:

2 $$E_{CT}=E_{K}+E_{C}+E_{P}$$

where *E*_*K*_ is the kinetic energy of the electron and hole delocalized across the donor–acceptor interface, *E*_*C*_ is the Coulomb interaction energy between the electron and hole, and *E*_*P*_ is the stabilization energy due to polarization of the surrounding dielectric medium. These terms collectively describe the energetics of a bound electron–hole pair at the P3HT-IDTBR interface.

#### 2.2.1 Kinetic Energy

The kinetic energy of a charge carrier is computed as the expectation value of the tight-binding Hamiltonian (eq 1) for a given wavefunction. We define separate one-body Hamiltonians, *H* ^*e*^ and *H* ^*h*^, for the electron and hole, respectively, each delocalized over the donor and acceptor molecules. Within the tight-binding framework, the local electronic states are represented by the HOMOs and LUMOs of the constituent monomers or sites.

The total kinetic energy *E*_*K*_ of the CT exciton is given by

3 $$E_{K}=-\left\langle \psi^{h}\right|H^{h}\left|\psi^{h}\right\rangle +\left\langle \psi^{e}\right|H^{e}\left|\psi^{e}\right\rangle$$

Here, $\left|\psi^{h}\right\rangle =\left\{a_{i}^{h}\right\}$ and $\left|\psi^{e}\right\rangle =\left\{a_{i}^{e}\right\}$ are the hole and electron wavefunctions, represented as site amplitudes. The negative sign for the hole term reflects its interpretation as the absence of an electron.^44^ In the following, we refer to *E*_*K*_ as the sum of the one-body kinetic energies of the hole and electron in the CT state, and denote this contribution by *E*_1b_ ≡ *E*_*K*_.

For clarity, we also define the individual one-body kinetic energies in the CT state as $E_{1b,h}^{\mathrm{CT}}=-\left\langle \psi^{h}\right|H^{h}\left|\psi^{h}\right\rangle$ and $E_{1b,e}^{\mathrm{CT}}=\left\langle \psi^{e}\right|H^{e}\left|\psi^{e}\right\rangle$ , so that $E_{1b}=E_{1b,h}^{\mathrm{CT}}+E_{1b,e}^{\mathrm{CT}}=E_{K}$ .

The electron and hole Hamiltonians *H* ^*e*^ and *H* ^*h*^ at a P3HT-IDTBR interface contain onsite energies for each aromatic unit and hopping terms between neighboring sites. They include intrachain hopping along the IDTBR backbone and the P3HT chain, as well as a single interchain hopping element *t*_*ab*_ between the close-contact BT ring on IDTBR and the adjacent thiophene ring on P3HT.

Both intrachain and interchain hopping matrix elements *t*_*ij*_ depend on orbital overlap and therefore on the relative geometry of the monomers. The tight-binding parameters are locally transferable and are determined from DFT energies of monomers and dimers. Onsite energies are listed in Table 1, and hopping parameters are described in Section 2.3.

**Table 1:** Calculated Onsite Energies ( $\epsilon_{k}^{e}$ and $\epsilon_{k}^{h}$ ), and Coulomb Interaction Parameter (*E*_*s,k*_) and Smearing Length (*σ_k_*) for Constituent Monomers

Monomer	$\epsilon_{k}^{e}\left(\mathrm{eV}\right)$	$\epsilon_{k}^{h}\left(\mathrm{eV}\right)$	*E*_*s*,*k*_ (eV)	σ_*k*_ (Å)
Thiophene (Th)	1.71	–8.88	4.72	4.05
Phenylene (Ph)	1.91	–9.21	4.91	4.34
Benzothiadiazole (BT)	–0.6	–8.8	4.31	4.42
Rhodanine (Rh)	–0.9	–8.7	3.85	6.25

#### 2.2.2 Coulomb Energy Term

In the tight-binding model, the electron and hole wavefunction are expressed as linear combination of LUMO and HOMO orbitals of the constituent monomers. To estimate the Coulomb interaction energy *E*_*C*_,^39,44^ we approximated these orbitals as Gaussian charge distributions:

4 $$\rho \left(r\right)=q_{0}\frac{e^{r^{2}/{\left(2\sigma \right)}^{2}}}{{\left(2\pi \sigma \right)}^{3/2}}$$

where σ is the characteristic smearing length and *q*_0_ is the elementary charge. The Gaussian width or characteristic smearing length σ is extracted from DFT calculations of the charge distribution on monomer units.^44^ The direct Coulomb integral between two such charge distributions at sites *i* and *j* is

5 $$\begin{array}{ll} D_{ij} & =\int dr\int dr^{\prime }\frac{\rho_{i}\left(r\right)\rho_{j}\left(r^{\prime }\right)}{\left|r-r^{\prime }\right|}\\ & =\frac{1}{R_{ij}}erf\left(\frac{R_{ij}}{2\sigma_{ij}}\right) \end{array}$$

The total Coulomb interaction energy is then

6 $$E_{C}=-\underset{i}{\sum }{|a_{i}^{h}|}^{2}{|a_{i}^{e}|}^{2}E_{s,i}-\underset{i\neq j}{\sum }{|a_{i}^{h}|}^{2}{|a_{j}^{e}|}^{2}\frac{1}{R_{ij}}erf\left(\frac{R_{ij}}{2\sigma_{ij}}\right)$$

Here, $a_{i}^{e}$ and $a_{i}^{h}$ are the wavefunction amplitudes for the electron and hole at site *i*, *E*_*s*,*i*_ is the onsite Coulomb interaction parameter obtained by fitting to DFT and TDDFT results for monomers^39,44^ *R*_*ij*_ is the distance between sites *i* and *j*; and σ_*ij*_ = (σ_*i*_ + σ_*j*_)/2 is the average smearing length. Table 1 lists the relevant parameters for each monomer.

The first term in eq 6 accounts for onsite Coulomb interactions, while the second term captures offsite direct Coulomb interactions. Offsite exchange contributions, which scale as $1/R_{ij}^{3}$ , are relatively small and thus neglected.

#### 2.2.3 Polarization Energy

In a dielectric medium, the presence of a charge carrier, either an electron or a hole, induces a polarization response in the surrounding material. This response manifests as a redistribution of bound charges that partially screen the electric field of the explicit charge distribution. The resulting stabilization contributes to the total energy of the charge transfer (CT) exciton and is captured by the polarization energy term *E*_*P*_.

As illustrated in Figure 2, each monomer site *i* carries an explicit charge *q*_*i*_, derived from the difference in occupation probabilities of the hole and electron wavefunctions. These charges induce local polarization vectors *P*_*i*_, which oppose the field of the explicit charges and reduce the overall electrostatic energy of the system.

**Figure 2:**
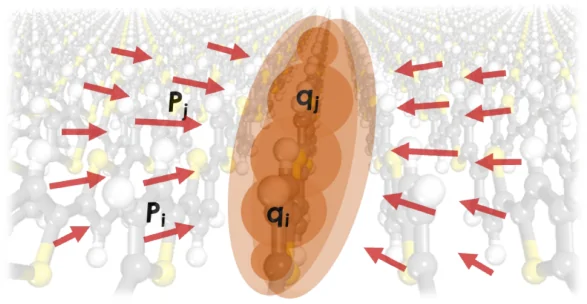
Schematic representation of a charge carrier and its induced polarization in the surrounding dielectric medium. Red arrows indicate polarization vectors (*P_i_*) induced by explicit charges (*q_i_*) localized on monomer sites.

In a continuous dielectric medium characterized by a relative permittivity ϵ_*r*_, the induced polarization charge density ρ_*P*_ is related to the explicit charge density ρ_*f*_ by

7 $$\rho_{P}=-\left(1-\frac{1}{\epsilon_{r}}\right)\rho_{f}$$

The total electrostatic energy of the system, including interactions between explicit charges and induced polarization, is given by

8 $$E_{static}=E_{qq}+E_{qp}+E_{pp}$$

where *E*_*qq*_ is the charge–charge interaction energy, *E*_*qp*_ is the charge-polarization interaction energy, and *E*_*pp*_ is the polarization-polarization interaction energy. *E*_*pp*_ is quadratic in the polarization amplitude *P*_*i*_, while *E*_*qp*_ is linear in the polarization amplitude. Thus, minimizing *E*_*static*_ with respect to the polarization amplitudes yields the relation *E*_*qp*_ = −2*E*_*pp*_, leading to *E*_*static*_ = *E*_*qq*_ – *E*_*pp*_ and *E*_*static*_ = *E*_*qq*_/ϵ_*r*_.

From this, the polarization energy *E*_*P*_, defined as the energy gain due to the dielectric response, is expressed as

9 $$E_{P}=E_{pp}+E_{qp}=-\left(1-\frac{1}{\epsilon_{r}}\right)\frac{1}{2}\underset{i,\;j}{\sum }q_{i}q_{j}E_{qq}\left(i,j\right)$$

Here, $q_{i}=e\left({|a_{i}^{h}|}^{2}-{|a_{i}^{e}|}^{2}\right)$ is the net charge on site *i*, and *E*_*qq*_(*q*_*i*_, *q*_*j*_) is the Coulomb interaction between charges at sites *i* and *j*, modeled as 3D Gaussian distributions:

10 $$E_{qq}\left(q_{i},\;q_{j}\right)=\frac{1}{R_{ij}}erf\left(\frac{R_{ij}}{2\sigma_{ij}}\right)$$

with *R*_*ij*_ = |*r*_*i*_ – *r*_*j*_| and $\sigma_{ij}=\frac{\sigma_{i}+\sigma_{j}}{2}$ . The polarization energy *E*_*P*_ is inherently negative, reflecting the reduction in electrostatic repulsion and the overall stabilization of the CT exciton energy in a polarizable medium.

Combining eqs 2, 3, 6, and 9, the total CT exciton energy is

11 $$\begin{array}{ll} E_{CT}= & -\left\langle \psi^{h}\right|H^{h}\left|\psi^{h}\right\rangle +\left\langle \psi^{e}\right|H^{e}\left|\psi^{e}\right\rangle \\ & -\underset{i}{\sum }{|a_{i}^{h}|}^{2}{|a_{i}^{e}|}^{2}E_{s,i}-\underset{i\neq j}{\sum }{|a_{i}^{h}|}^{2}{|a_{j}^{e}|}^{2}\frac{1}{R_{ij}}erf\left(\frac{R_{ij}}{2\sigma }\right)\\ & -\left(1-\frac{1}{\epsilon_{r}}\right)\frac{1}{2}\underset{i,j}{\sum }\left({|a_{i}^{h}|}^{2}-{|a_{i}^{e}|}^{2}\right)\left({|a_{j}^{h}|}^{2}-{|a_{j}^{e}|}^{2}\right)\frac{1}{R_{ij}}erf\left(\frac{R_{ij}}{2\sigma_{ij}}\right) \end{array}$$

To predict the optimal charge-transfer exciton at the donor–acceptor interface, we minimize the total exciton energy *E*_CT_ with respect to the onsite amplitudes of the electron $\left(a_{i}^{e}\right)$ and hole $\left(a_{i}^{h}\right)$ . We allow both electron and hole to have amplitude on both donor and acceptor sites such that localized and CT excitons naturally emerge from minimizing *E*_CT_.

### 2.3 Donor–Acceptor Interface

To model charge transfer (CT) excitons at the donor–acceptor interface between P3HT and O-IDTBR, realistic atomistic configurations of the donor–acceptor blend are essential. We employed a well-equilibrated morphology of an amorphous P3HT:IDTBR blend, generated using molecular dynamics (MD) simulations. The simulated melt contains 128 P3HT chains, each with 24 monomers, and 300 O-IDTBR molecules confined within a 7 × 7 × 27.5 nm^3^ simulation box under periodic boundary conditions. To accelerate equilibration, Agarwala et al. used a virtual-site coarse-graining approach, allowing the system to equilibrate over 2 μs.^46^ Figure 3 displays a snapshot of the equilibrated interface.

**Figure 3:**
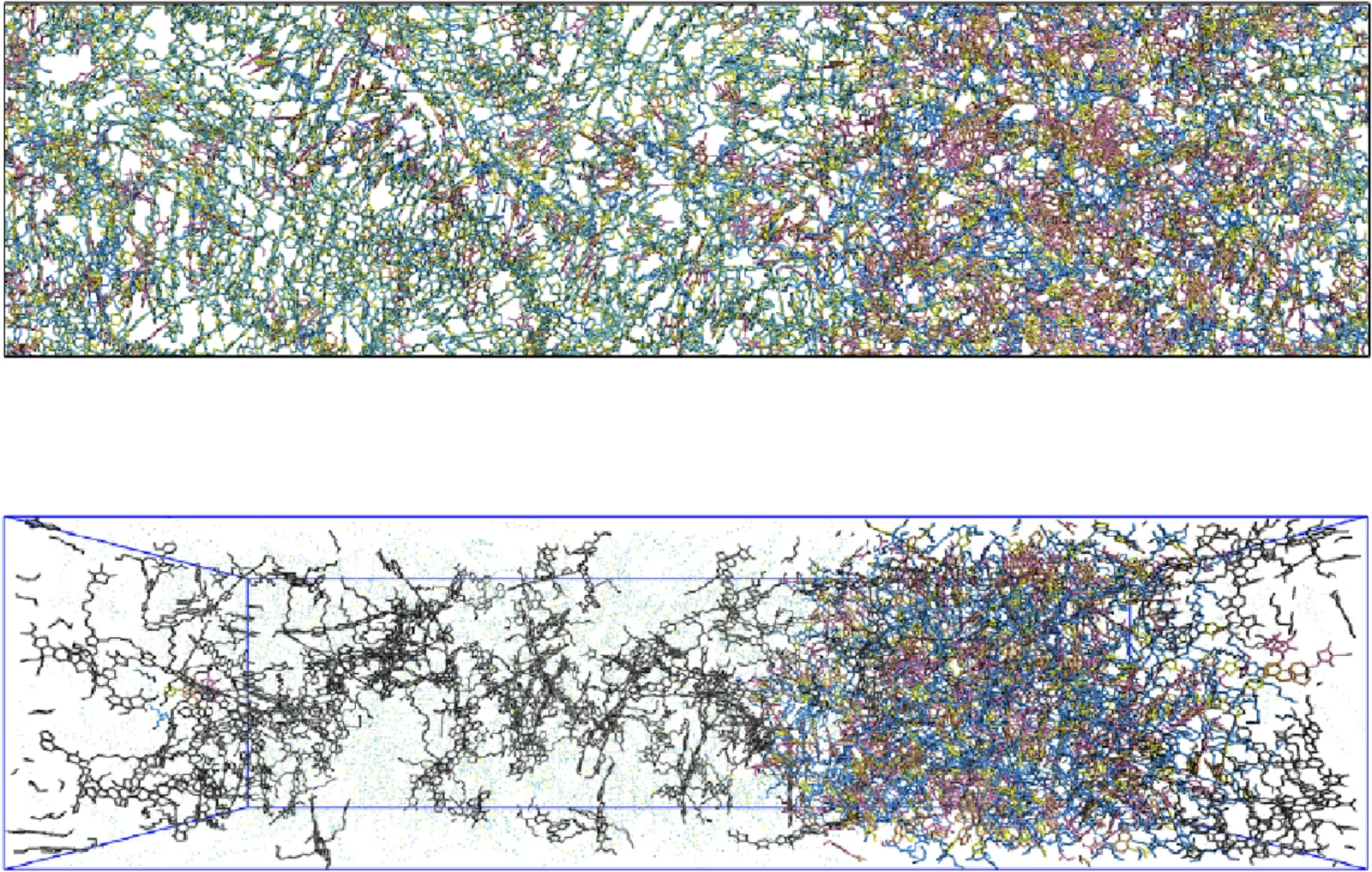
A well-equilibrated blend of poly­(3-hexylthiophene) (P3HT) chains and O-IDTBR obtained using MD simulation, showing both P3HT and O-IDTBR (top) and O-IDTBR only (bottom).

To identify potential sites for CT exciton formation, we analyzed close contacts between P3HT chains and IDTBR molecules within the blend morphology. The most probable contact occurred between the benzothiadiazole (BT) ring of O-IDTBR and the thiophene ring of P3HT, attributed to steric hindrance from the octyl side chain on the indacenodithiophene (IDT) unit of IDTBR. We identified all BT-thiophene pairs across different molecules with center-to-center distances less than 5 Å. In total, 311 close contacts were found between P3HT and O-IDTBR. After excluding pairs where the thiophene unit was located within three monomers of either end of the P3HT chain, the number of relevant contacts was reduced to 237.

To describe CT exciton at donor–acceptor interface, we focused on IDTBR molecules and P3HT chain segments consisting of seven thiophenes units identified to be in close contact. Each segment includes a central thiophene ring in proximity to a BT ring of an IDTBR molecule, flanked by three thiophene rings on either side. The interaction parameter between the central thiophene on the P3HT chain segment and the close-contact BT ring on IDTBR is characterized by a hopping matrix element *t*_*ab*_, which enables charge carriers to hop between successive moieties, and spread out over chain segments and molecules.

#### 2.3.1 Hopping Matrix Parameters

We calculate the hopping matrix elements along P3HT chains and IDTBR molecules using DFT applied to constituent monomers and the various dimers they can form. To determine the hopping matrix element *t* between two bonded monomers, we compute the DFT energies of anionic, cationic and neutral ground states for each monomer and for the codimer formed by consecutive monomers taking into account their molecular geometries.^44^ All DFT calculations were performed using the B3LYP functional and the 6–311g­(d) basis set.

Using these DFT energies, we evaluate *t* between adjacent monomers for all P3HT chain segments and IDTBR molecules identified to be in close contact, based on molecular structures extracted from the equilibrated MD trajectories.

Figure 4 shows the distribution of intrachain hopping matrix elements along P3HT chains for both hole $\left(t_{Th}^{h}\right)$ and electron $\left(t_{Th}^{e}\right)$ . The dihedral angles between consecutive thiophene units in P3HT are flexible and can rotate freely, resulting in significant dihedral disorder. This conformational variability leads to a broad distribution of intrachain hopping matrix elements for both electrons and holes along the P3HT backbone.

**Figure 4:**
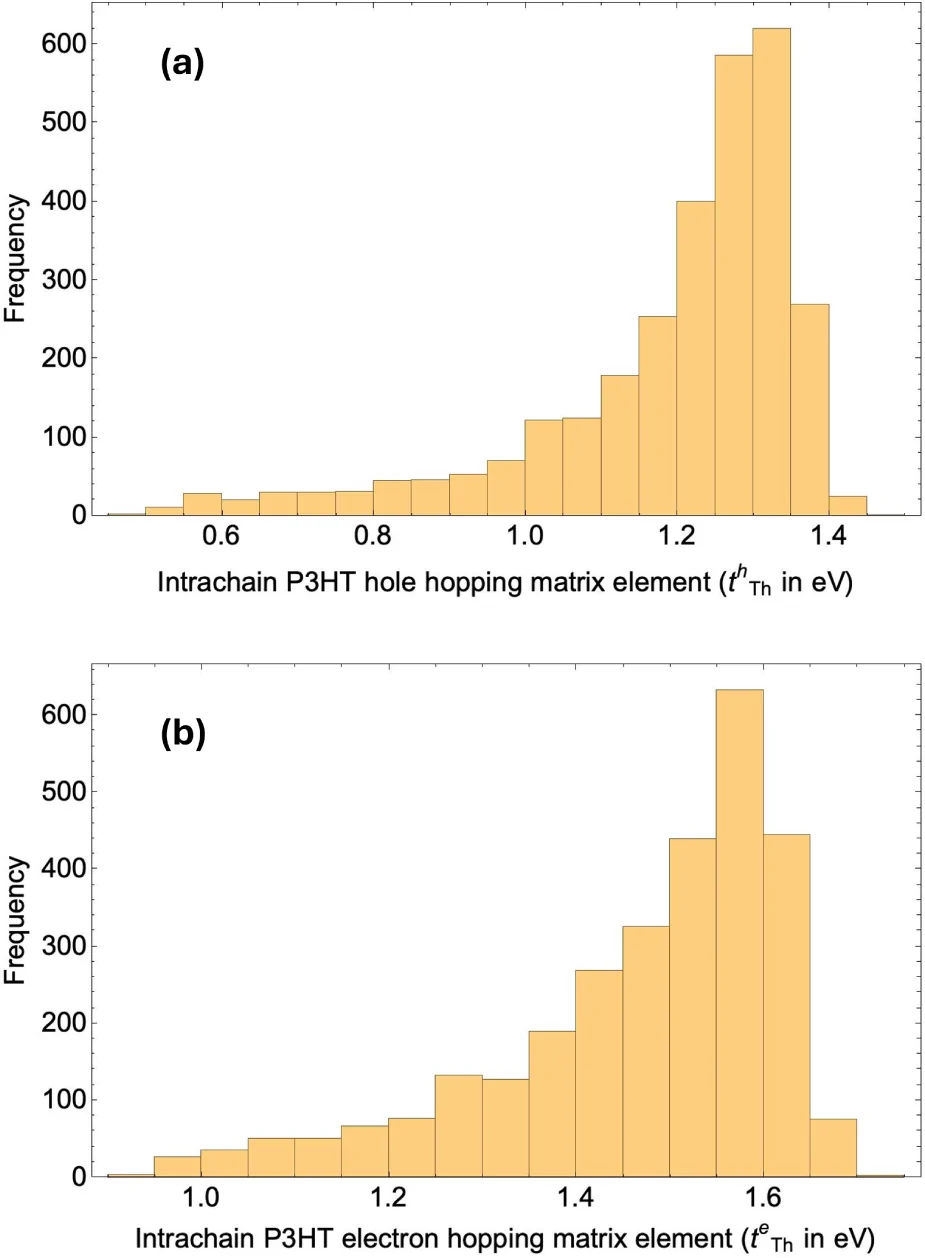
Distribution of intrachain hopping matrix elements along P3HT for (a) hole transport $\left(t_{Th}^{h}\right)$ , and (b) electron transport $\left(t_{Th}^{e}\right)$ .

Two thiophene rings are bridged on either side of a phenyl ring to form the indacenodithiophene (IDT) core of an IDTBR molecule. The hopping matrix elements between the thiophene and phenylene rings are calculated to be $t_{PhTh}^{h}=1.28\mathrm{eV}$ and $t_{PhTh}^{e}=1.31\mathrm{eV}$ . These values are treated as constant because the dihedral angle between these two rings is fixed and does not rotate.

Figure 5 shows the distribution of hopping matrix elements between thiophene-BT and BT-rhodanine units for both hole and electron delocalization in IDTBR that are in close contact with P3HT. Table 2 lists the mean and standard deviation of intrachain hopping matrix elements in P3HT chains and IDTBR molecules, corresponding to the distributions shown in Figures 4 and 5. Compared to P3HT chains, IDTBR molecules exhibit a narrower distribution (lower standard deviation) of hopping matrix elements, indicating a more rigid and linear molecular structure.

**Figure 5:**
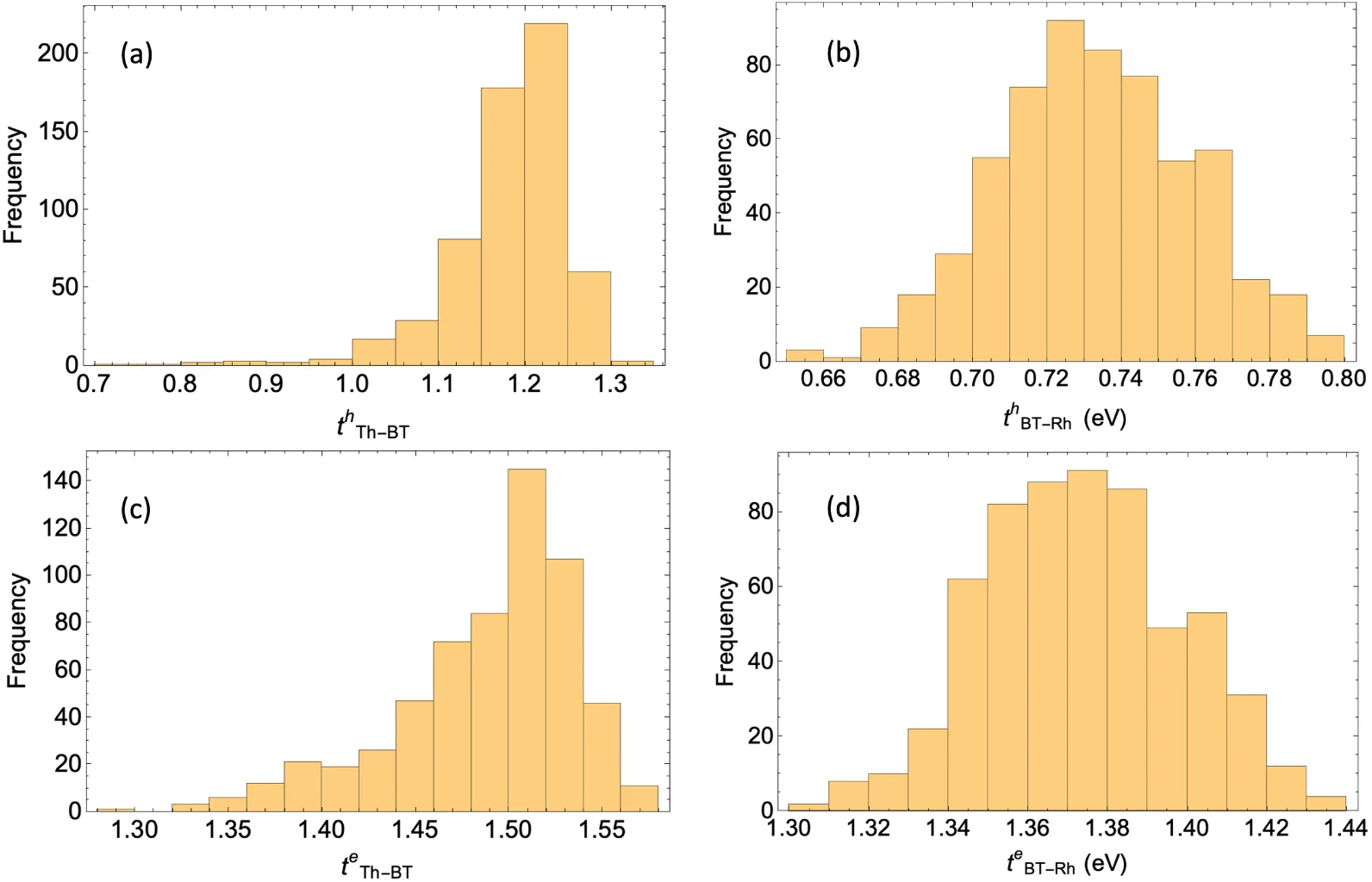
Distribution of intrachain hopping matrix elements for IDTBR: (a) hole hopping between thiophene and benzothiadiazole $\left(t_{ThBT}^{h}\right)$ , (b) hole hopping between benzothiadiazole and rhodanine $\left(t_{BTRh}^{h}\right)$ , (c) electron hopping between thiophene and benzothiadiazole $\left(t_{ThBT}^{e}\right)$ , and (d) electron hopping between benzothiadiazole and rhodanine $\left(t_{BTRh}^{h}\right)$ .

**Table 2:** Intrachain Hopping Matrix Element Values for P3HT Chains and IDTBR Molecules Computed from Distributions in Figures 4 and 5

Hopping matrix element (t)	Value for hole (eV)	Value for electron (eV)
P3HT Thiophene-Thiophene	$t_{Th}^{h}=1.19\pm 0.18$	$t_{Th}^{e}=1.47\pm 0.15$
IDTBR Phenylene-Thiophene	$t_{RhTh}^{h}=1.28$	$t_{RhTh}^{e}=1.31$
IDTBR Thiophene-Benzothiadiazole	$t_{ThBT}^{h}=1.18\pm 0.07$	$t_{ThBT}^{e}=1.49\pm 0.05$
IDTBR Benzothiadiazole-Rhodanine	$t_{BTRh}^{h}=1.37\pm 0.025$	$t_{BTRh}^{e}=0.73\pm 0.026$

To calculate the interchain hopping matrix element for hole transport (*t*_*ab*_) between an IDTBR molecule and a P3HT chain in close contact, we evaluate the electronic coupling between the thiophene and benzothiadiazole (BT) rings using a previously developed machine learning (ML) model.^47^ This model estimates the orbital overlap between the HOMOs of two nonbonded rings based on their relative geometry.

The same approach can be extended in principle to compute the orbital overlap between LUMOs, allowing us to estimate the interchain hopping matrix elements for electron delocalization at the IDTBR-P3HT interface. However, in this work, we assume for simplicity that the interchain hopping matrix elements for hole and electron are equal.

The resulting distribution of *t*_*ab*_ values, extracted from all identified close contacts in the MD trajectory, is shown in Figure 6. These values are used to construct the tight-binding Hamiltonian across the donor–acceptor interface and to evaluate the CT exciton energetics.

**Figure 6:**
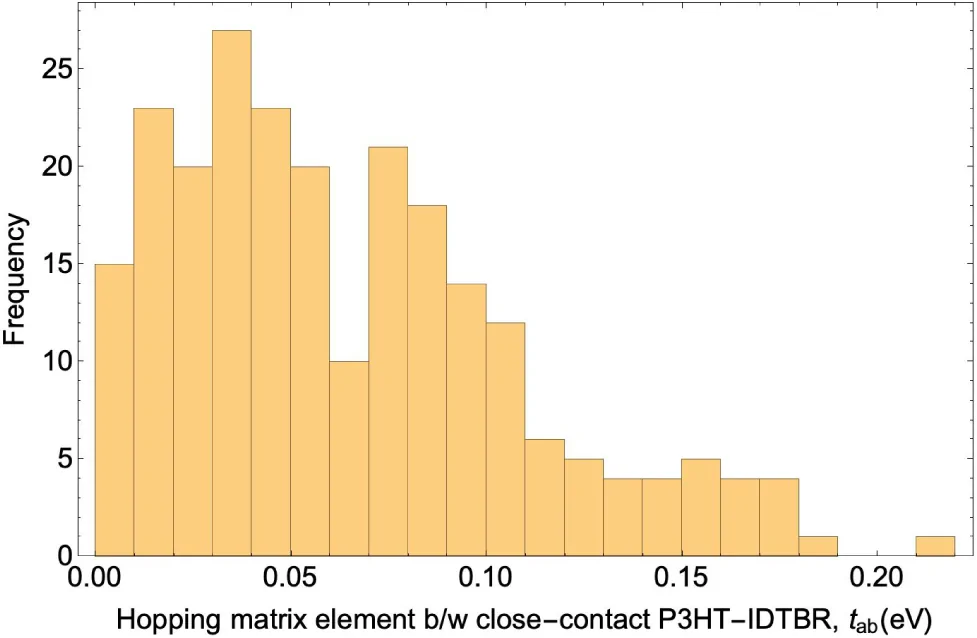
Distribution of interchain hopping matrix elements (*t_ab_*) between P3HT and O-IDTBR close contacts in a well-equilibrated amorphous P3HT:IDTBR blend.

## 3 Results and Discussion

### 3.1 Charge-Transfer Exciton at the Interface

To evaluate the energetics and spatial structure of charge-transfer (CT) excitons, we minimize the total exciton energy with respect to the onsite amplitudes of the electron $\left(a_{i}^{e}\right)$ and hole $\left(a_{i}^{h}\right)$ across each donor–acceptor pair identified in the MD trajectory. The electron and hole wavefunctions are allowed to delocalize over the P3HT chain segment and the IDTBR molecule in close contact, enabling the model to capture both localized and charge-separated configurations.

We first consider an idealized interface to illustrate how the exciton energy varies as the electron is transferred from P3HT to IDTBR. The model system consists of a planar IDTBR molecule and a P3HT chain oriented perpendicularly at a stacking distance of 4.5 Å, with the BT unit on IDTBR stacked face-on with a thiophene ring on P3HT (see Figure 1). Starting with both the hole and electron wavefunctions fully localized on P3HT, we gradually allow the electron to leak onto IDTBR via a hopping matrix element *t*_*ab*_ = 0.1 eV between close-contact BT and thiophene rings. This defines a fractional occupancy *f* of the electron on IDTBR, while the hole remains fixed on the P3HT segment. For each value of *f*, we minimize the total exciton energy with respect to the wavefunction amplitudes, subject to the constraint that the hole remains entirely on P3HT and the electron fraction on IDTBR is fixed.

Figure 7 shows the resulting exciton energy as a function of electron fraction *f* on IDTBR. The value *f* = 0 corresponds to a fully localized exciton on P3HT, while *f* = 1 represents a fully separated CT exciton with the electron on IDTBR and the hole on P3HT. For this idealized configuration, the energy landscape is downhill as the electron moves from P3HT to IDTBR, indicating a thermodynamic preference for charge separation at this interface.

**Figure 7:**
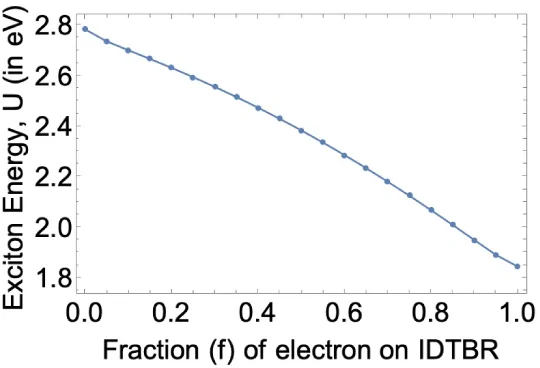
Exciton energy as a function of electron fraction on IDTBR for an idealized P3HT-IDTBR interface geometry (Figure 1).

We next examine the ensemble of P3HT-IDTBR close contacts identified from the simulated amorphous blend. For each configuration, we locate two local minima: an excitons fully localized on IDTBR, and a CT states with electron–hole separation across the interface. Figures 8 and 9 present the corresponding energy distributions. Both types of excitons are observed, and their relative stability varies across configurations: in some cases the IDTBR-localized exciton is energetically favored, while in others the CT exciton is more stable. This variability reflects the sensitivity of exciton energetics to the local molecular arrangement at the donor–acceptor interface.

**Figure 8:**
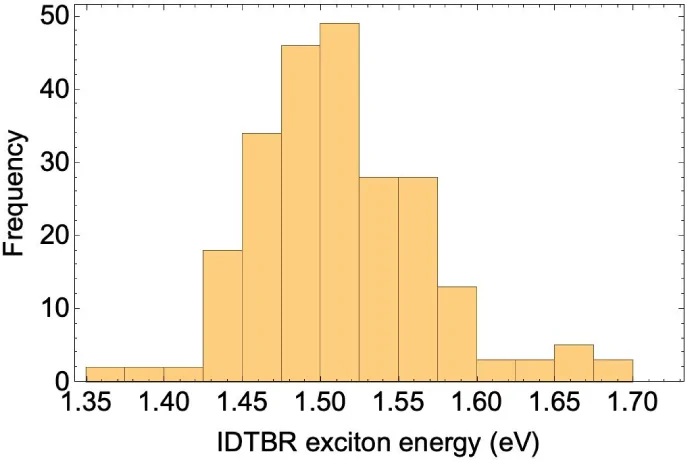
Energy distribution of exciton localized on IDTBR across all P3HT-IDTBR close contacts.

**Figure 9:**
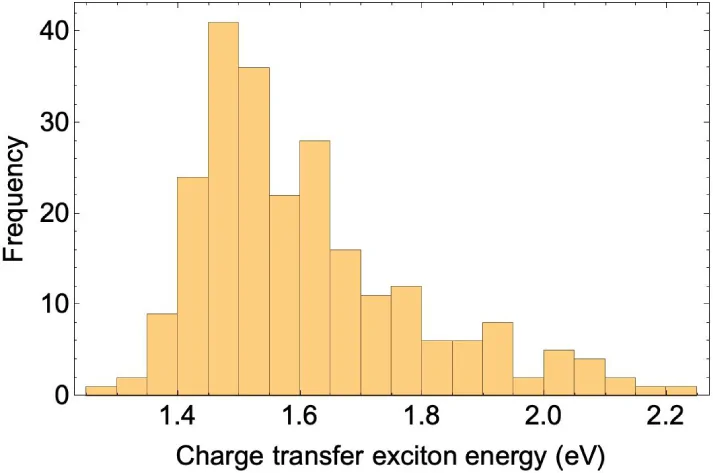
Energy distribution of CT exciton across all P3HT-IDTBR close contacts.

Figure 10 compares the CT exciton energy with the corresponding IDTBR-localized exciton energy for each donor–acceptor configuration. Data points above the 45° red dashed line indicate configurations where the CT exciton is more favorable, while points below the line corresponds to cases where IDTBR exciton is more stable. The broad spread of points confirms that the energy landscape for charge transfer is heterogeneous: depending on the local geometry, the lowest-energy exciton may be either localized on IDTBR or delocalized across the interface.

**Figure 10:**
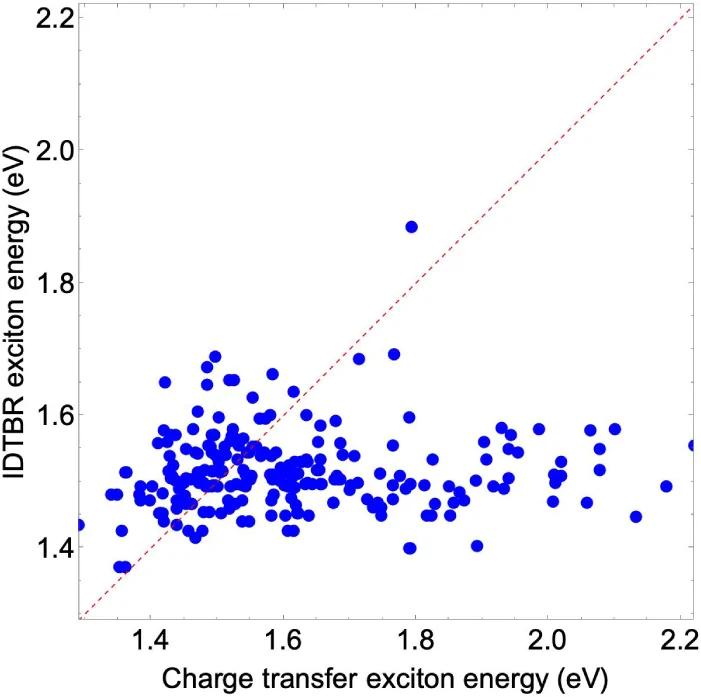
Scatter plot comparing CT exciton energy and IDTBR exciton energy for each donor–acceptor configuration. The red dashed line indicates equal energy.

To identify what aspects of the interfacial configuration control this heterogeneity, we considered two simple hypotheses. First, “good polarons make good CT excitons”: low-energy CT states form between straight, low-energy polaron segments. Second, “close polarons make good CT excitons”: low-energy CT states are additionally stabilized by strong electron–hole Coulomb attraction between closely aligned segments.

To test these ideas, we decomposed the CT exciton energy *E*_CT_ for each configuration into a one-body contribution *E*_1b_ (the sum of electron and hole kinetic energies $E_{1b,e}^{\mathrm{CT}}+E_{1b,h}^{\mathrm{CT}}=E_{K}$ ) and a two-body contribution *E*_int_ = *E*_C_ + *E*_P_ (Coulomb plus dielectric polarization stabilization). We then constructed scatter plots of *E*_CT_ versus *E*_1b_ and versus *E*_int_.

The CT exciton energy exhibits a strong, nearly linear correlation with the one-body contribution (Figure 11), whereas its dependence on the Coulomb + polarization term is much weaker and more scattered (Figure 12). This indicates that the dominant source of CT-energy variation across configurations is the underlying polaron energy landscape, controlled primarily by intrachain dihedral disorder and local conjugation. In this sense, “good polarons make good CT excitons”: straight, low-energy segments on P3HT and IDTBR produce systematically lower CT energies.

**Figure 11:**
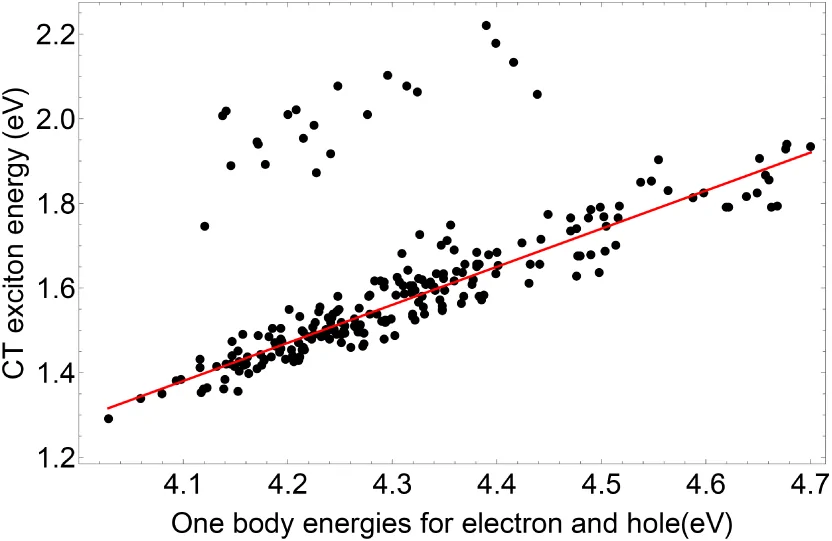
CT exciton energy versus sum of one-body kinetic energies of electron and hole in CT state (*E*_1b_) for all P3HT-IDTBR interfaces. The red line shows a linear least-squares fit.

**Figure 12:**
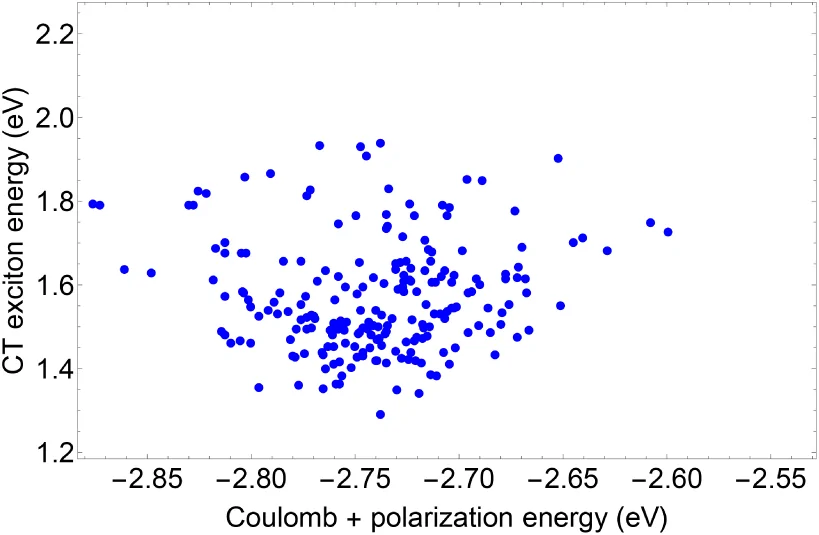
CT exciton energy versus Coulomb + polarization interaction energy for all P3HT-IDTBR interfaces.

The one-body correlation nevertheless contains a distinct subset of outliers with unusually high CT energies, which appear as points well above the main trendline in Figure 11. Inspection of these configurations shows that they correspond to states in which the exciton is significantly more stable when localized on IDTBR than when charge-separated, with *E*_CT_ – *E*_IDTBR_ typically in the range 0.3–0.7 eV. That is, they correspond to points far to the right in Figure 10. Energetically, these outlier configurations would be more stable as excitons residing exclusively on the IDTBR acceptor molecule; however, we have effectively forced the charges to separate to the donor strand and acceptor molecule, whereupon the Coulomb stabilization is weak and the dielectric polarization stabilization is strong—that is, the configuration begins to resemble truly separated carriers, rather than a CT exciton.

Table 3 summarizes the mean Coulomb, polarization, and total interaction energies for the main population versus the outlier group. The main group shows substantially stronger Coulomb stabilization and a more favorable net *E*_C_ + *E*_P_, whereas the outliers are characterized by weak Coulomb attraction partially compensated by larger polarization stabilization. These states are better described as spatially separated electron and hole polarons than as tightly bound interfacial CT excitons, and we therefore exclude them when analyzing trends within the CT states.

**Table 3:** Mean Coulomb, Polarization, and Total Interaction Energies for CT Excitons on the Main One-Body Trendline and for Outlier Configurations

Group	Mean Coulomb (eV)	Mean polarization (eV)	Mean Coulomb + Pol (eV)
Main	–2.31	–0.43	–2.74
Outliers	–0.95	–1.30	–2.24

After removing these outliers, the correlation between *E*_CT_ and the one-body kinetic energy *E*_1b_ becomes even tighter, reinforcing the conclusion that the primary control parameter for CT energetics is the local polaron geometry.

To assess whether Coulomb attraction nevertheless contributes to the remaining scatter, we subtracted the best-fit linear relation between *E*_CT_ and *E*_1b_ and plotted the residual energy Δ*E*_CT_ versus the Coulomb + polarization term. The residuals show a clear secondary correlation (Figure 13), with more negative interaction energies systematically lowering *E*_CT_ by tens of meV. Thus, while good polarons set the main energetic ordering of CT states, close polarons which have strong electron–hole Coulomb interaction and more dielectric stabilization, make better CT excitons by providing additional stabilization on a smaller energy scale.

**Figure 13:**
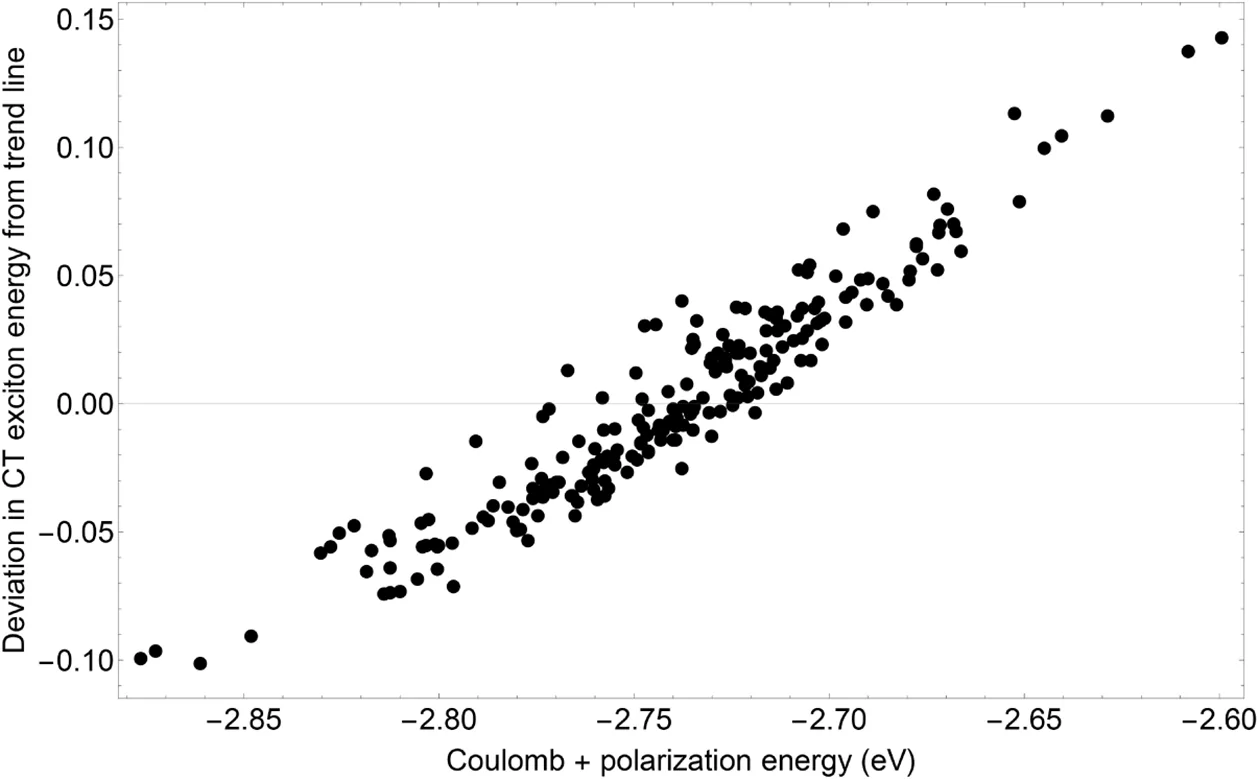
Residual CT exciton energy (deviation from the best-fit one-body trend) versus Coulomb + polarization interaction energy, after excluding outliers.

These results link the broad CT energy distribution in Figures 8 and 9 directly to structural features at the P3HT-IDTBR interface: intrachain conformation determines the primary CT energy scale through the polaron landscape, while variations in interchain separation and orientation modulate the energy via Coulomb and polarization interactions.

### 3.2 Energy Landscape for CT Exciton Formation and Dissociation

To place the CT excitons energetics in a broader context, we compare the computed CT energies with the HOMO–LUMO gaps, bulk exciton energies, and polaron energies of P3HT and IDTBR. Figure 14 summarizes this progression, illustrating how photoexcitation leads to localized excitons, followed by interfacial charge transfer and eventual dissociation into free carriers. The energy levels shown are extracted from our tight-binding model on planar molecular configurations and from prior work on polaron transport and exciton binding in conjugated polymers.^27,40,43,44^

**Figure 14:**
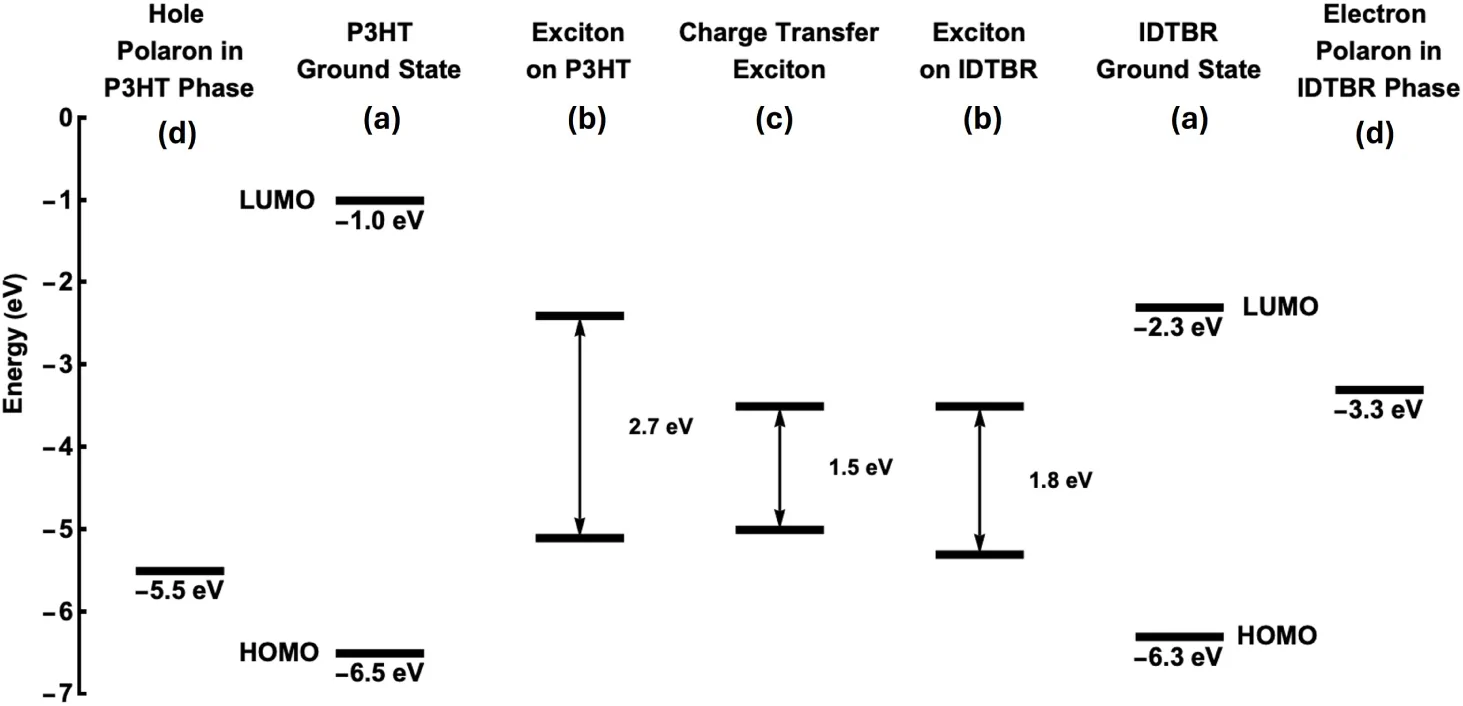
Energy diagram comparing (a) ground-state HOMO and LUMO levels, (b) bulk exciton energies, (c) CT exciton energy at interface, and (d) polaron energies for P3HT-IDTBR system.

The analysis in the last section shows that the broad distribution of CT exciton energies is governed primarily by the underlying polaron energy landscape, with Coulomb and polarization interactions providing an additional stabilization that explains the scatter about this trend. Here we focus on how the CT states fit into the overall energy landscape relative to bulk excitons and free polarons. In this framework, the CT exciton energy lies between the bulk exciton and polaron energies, consistent with its role as an intermediate in the charge separation pathway.

To quantify the dissociation potential, we define the CT exciton stabilization energy relative to separated polarons as

12 $$E_{stabilization}^{CT}=E_{CT}-\left(E_{elec.\;polaron}^{IDTBR}+E_{hole\;polaron}^{P3HT}\right)$$

By this convention, negative values of $E_{stabilization}^{CT}$ indicate that the CT state is energetically below, and therefore bound with respect to, a pair of noninteracting polarons. Using our previously validated tight-binding model for polaron hopping, the hole polaron energy in P3HT is approximately 1.0 eV, and the electron polaron energy in IDTBR is around 1.0 eV, yielding a total free-carrier energy of ∼2.0 eV. Across the ensemble of P3HT-IDTBR interfaces, most CT exciton energies fall between 1.6 and 1.8 eV, resulting in CT stabilization energies in the range of −0.2 to −0.4 eV.

This modest binding is consistent with the picture developed in the last section: intrachain geometry tunes the primary CT energy scale to lie only a few hundred meV below the free-polaron limit, while Coulomb and polarization interactions provide additional, configuration-dependent stabilization without creating excessively deep traps. In a functioning device, interfaces that produce such shallowly bound CT states relative to polaron energies are more likely to yield efficient charge separation. Deeply bound CT states, by contrast, may localize carriers and reduce power-conversion efficiency. Our results suggest that P3HT-IDTBR interfaces strike a favorable balance, with CT stabilization energies tuned by morphology and electronic structure to promote dissociation without compromising formation.

## 4 Conclusions

In this work, we developed a multiscale framework to model charge-transfer (CT) excitons at donor–acceptor interfaces in organic semiconductors using P3HT-IDTBR as a representative system. By extending our previously established tight-binding model to include Coulomb and polarization effects, we computed CT exciton energies and wave functions across hundreds of realistic interfacial configurations extracted from molecular dynamics simulations. All electronic parameters were derived from first-principles DFT based on local molecular geometries, allowing us to isolate the role of structural heterogeneity in shaping exciton behavior.

Our results reveal that the energetics of CT excitons are highly sensitive to local molecular arrangement, but this sentivity is not random. The dominant contribution to the CT energy variation arises from the one-body kinetic energy landscape, which is controlled by intrachain dihedral disorder and local conjugation. Within this landscape, “good polarons make good CT excitons”: straight, low-energy segments on P3HT and IDTBR form the lowest-energy CT states. Coulomb attraction and dielectric polarization then provide an additional, smaller-scale stabilization that explains much of the scatter around the primary one-body trend.

Placing these results in an energy-level diagram, we find that the CT exciton energies lie between the bulk exciton and polaron energies, with typical stabilization of only a few hundred meV relative to free carriers. This shallow stabilization is consistent with CT excitons acting as intermediates in the charge-separation pathway rather than deep traps, and it rationalizes why P3HT-IDTBR interfaces can support efficient charge separation. Together, the one-body and two-body analyses suggest two complementary design principles: promote straight, well-conjugated donor and acceptor segments to form low-energy polarons, and ensure sufficiently close donor–acceptor contact to provide additional Coulomb and polarization stabilization without creating overly deep CT states.

While our model captures key physical contributions to CT exciton energetics, it does not yet include dynamic effects such as vibronic coupling, temperature-dependent disorder, or nonadiabatic transitions. Additionally, the dielectric environment is treated as homogeneous, whereas real devices may exhibit spatially varying screening. Future work can incorporate time-dependent simulations and explicit treatment of dielectric heterogeneity to better capture exciton dynamics and dissociation pathways. Extending this framework to other donor–acceptor systems and validating predictions against ultrafast spectroscopy will further enhance its utility for materials design. By connecting molecular identity, geometry, and electronic structure to exciton energetics, this work lays the foundation for predictive modeling of charge separation in organic photovoltaics.

## Data Availability

The data underlying this study are available at http://github.com/JinVish/ct-exciton-paper. The repository contains the computational input files, representative output files, processed data, and Mathematica scripts used for data analysis and generation of the reported results. All materials required to reproduce the key findings of this work are publicly accessible through the repository.
